# Atypical Hemolytic Uremic Syndrome Associated With Clostridium Difficile Infection

**DOI:** 10.7759/cureus.9005

**Published:** 2020-07-05

**Authors:** Qasim Khurshid, Anas Mahmoud, Maria Shahid, Alaa Mohamed, Amir Shahbaz

**Affiliations:** 1 Internal Medicine, Xinjiang Medical University, Urumqi, CHN; 2 Internal Medicine, Icahn School of Medicine at Mount Sinai, Queens General Hospital, New York, USA; 3 Internal Medicine, Chandka Medical College, Larkana, PAK; 4 Internal Medicine, Memorial Hermann Medical Center, Houston, USA; 5 Internal Medicine, California Institute of Behavioral Neurosciences & Psychology, Fairfield, USA; 6 Internal Medicine, Sheikh Zayed Hospital, Lahore, PAK; 7 Internal Medicine, Allama Iqbal Medical College, Lahore, PAK

**Keywords:** atypical hemolytic uremic syndrome, eculizumab

## Abstract

Atypical hemolytic uremic syndrome (aHUS), defines as non-Shiga toxin HUS, is thrombotic microangiopathy characterized by microangiopathic hemolytic anemia, consumptive thrombocytopenia, and renal impairment. aHUS is associated with high morbidity and mortality, necessitating the need for an early diagnosis to limit target organ damage. Mutations or autoantibodies against specific complement factors over‐activate the complement system forming microthrombi. aHUS has the potential to cause multi‐organ system dysfunction, but it predominantly affects the kidneys. aHUS is treated with eculizumab, a terminal blocker of the complement system. Clostridium difficile infection is a rare precipitant of aHUS. We present a case of aHUS associated with Clostridium difficile infection in a 60-year-old female patient that was successfully treated with eculizumab.

## Introduction

Thrombotic microangiopathy (TMA) is classified into three major categories including, Shiga toxin-producing *Escherichia coli* hemolytic uremic syndrome (STEC-HUS), thrombotic thrombocytopenic purpura, and atypical hemolytic uremic syndrome (aHUS) [1]. The majority of HUS cases are sporadic and triggered by infection, most notably certain strains of *E. coli* and other bacteria that produce toxic substances called Shiga-like toxins (SLTs). HUS most often affects children younger than 10 years and is not known to be associated with genetic mutations [1]. However, aHUS is usually due to a genetic or acquired defect in the regulation of complement activation on host cells [2]. The clinical presentation of aHUS and STEC‐HUS is similar. aHUS earned its name because it is not caused by either of the common etiological factors for typical HUS (Shiga toxin-produced by* E. coli* O157:H7 or *Shigella dysenteriae*) [3,4]. aHUS accounts for 5-10% of all documented cases of HUS and is associated with a poor prognosis [5]. It is a rare disease with an estimated incidence of 1 in 500,000 people per year in the United States. Triggers for the development of aHUS are more diverse and include drugs, complement regulation deficits, infections, and pregnancy [6]. In the literature, *Clostridium difficile* infection is described as a rare precipitant of aHUS [7-9].

## Case presentation

A 60-year-old female with a medical history of hypertension, which was well-controlled with lisinopril, presented to the emergency department with the complaint of multiple episodes of vomiting, profuse watery diarrhea, and decrease urine output for the last four days. One month before the admission, she had received ciprofloxacin for urinary tract infection. She appeared acutely ill with a blood pressure of 139/88 mmHg, a pulse of 100 beats/minute, a respiratory rate of 23 breaths/minute, and a temperature of 98°F. On physical examination, she had dry mucus membranes and yellow sclera. The abdomen was slightly distended with generalized tenderness but without guarding and rebound. The rest of the physical examination was unremarkable.

The blood work revealed the following: creatinine of 11.6 mg/dL (baseline creatinine was 1.3 mg/dL eight months ago), hemoglobin of 10.5 g/dL, hematocrit of 33%, reticulocytosis of 4.5 %, white cell count of 18.0 x 10^9^/L, and platelet count of 107 x 10^9^/L. The patient had an LDH (lactate dehydrogenase) level of 3,441 U/L and unconjugated bilirubin of 3.7 mg/dL. Peripheral blood smear showed a moderate number of schistocytes. Liver function tests and coagulation profile were within the normal range. Urine complete examination was positive for dysmorphic red blood cells and protein. Stool on the first day of admission was positive for C*. difficile* toxin A by enzyme immunoassay (EIA). Stool assay for SLT by EIA returned negative, and stool cultures were negative for *E. Coli* O157:H7 and other enteric pathogens. Antineutrophil cytoplasmic antibodies (ANCA), antinuclear antibodies (ANA), and double-stranded deoxyribonucleic acid (dsDNA) were negative. Serum folic acid and vitamin B12 levels were normal. Test results for hepatitis B surface antigen, hepatitis C antibody, human immunodeficiency virus (HIV), and coombs test were negative. Complement components were low, with C3 of 0.73 g/L (reference range [RR]: 0.85-1.60) and C4 of 0.08 g/L (RR: 0.12-0.36). The results are summarized in Table 1.

**Table 1:** Test results at presentation LDH, lactate dehydrogenase; BUN, blood urea nitrogen; ALT, alanine aminotransferase; AST, aspartate aminotransferase; ALP, alkaline phosphatase; PT, prothrombin time; APTT, activated partial thromboplastin time; INR, internal normalized ratio; C3 complement component 3; C4, complement component 4

Tests	Result	Reference Range
Hemoglobin	10.5 g/dL	12-15 g/dL
Hematocrit	33%	40-52%
Reticulocytes	4.5 %	0.5-1.5%
White cell count	18.0 x 10^9^/L	4-10 x 10^9^/L
Platelet count	107 x 10^9^/L	150-400 x 10^9^/L
LDH	3441 U/L	50-150 U/L
Unconjugated bilirubin	3.7 mg/dL	0.2-1.1 mg/dL
Creatinine	11.6 mg/dL	0.8-1.3 mg/dL
BUN	37 mg/dL	8-21 mg/dL
ALT	31 IU/L	5-42 IU/L
AST	37 IU/L	5-40 IU/L
ALP	89 IU/L	50-150 IU/L
PT	12 seconds	11-14 seconds
APTT	18 seconds	20-40 seconds
INR	1.1	0.9-1.2
C3	0.73 g/L	0.85-1.60 g/L
C4	0.08 g/L	0.12-0.36 g/L

Renal ultrasound revealed normal-sized kidneys with grade one echogenicity. Liver ultrasound was normal without biliary tract obstruction. Intravenous metronidazole and oral vancomycin started for *C. difficile*. Nephrology and hematology consultation was sought, and the multidisciplinary team decided to start plasmapheresis for a probable aHUS. ADAMTS13 (a disintegrin and metalloproteinase with a thrombospondin type 1 motif, member 13) activity levels were noted before the initiation of plasmapheresis. ADAMTS13 activity was 71%, which is within the normal reference range. We did not perform genetic studies due to limited resources.

**Figure 1:**
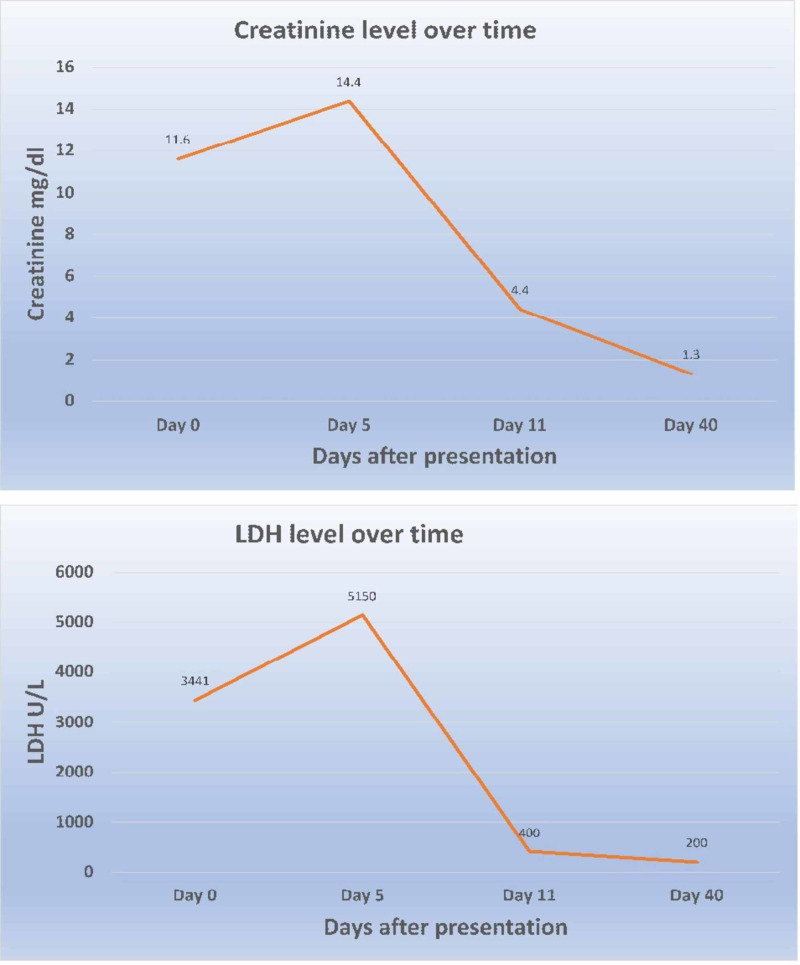
Graphical representation of serum creatinine (top) and serum LDH (bottom) over time from presentation until recovery. LDH, lactate dehydrogenase

We made a diagnosis of aHUS in the absence of STEC infection with regular ADAMTS13 activity. The patient underwent plasmapheresis and hemodialysis for seven days in the intensive care unit. During the hospital stay, her creatinine peaked at 14.4md/dL, and the nadir of her platelet count was 61,000/mm^3^. She received eculizumab infusion on the 9th day of hospital admission. She gradually improved, and at the time of discharge her LDH was 400 U/L and the creatinine level was 4.4 mg/dL. The graph of the time course of the patient's creatinine level and LDH is shown in Figure 1.

She was prescribed eculizumab upon discharge according to standard protocol. She followed up in the outpatient clinic after four weeks. Eculizumab treatment led to normalization in hemoglobin, platelet count, LDH, and creatinine levels.

## Discussion

TMA presents with thrombocytopenia, nonimmune microangiopathic hemolytic anemia, and end-organ damage [5]. Small vessel occlusion leads to hypoxia and ischemic end-organ damage, and the endothelial injury leads to platelet activation that causes platelet aggregation and thrombus formation. Acute kidney injury is the most common presentation due to the propensity of glomerular circulation to endothelial damage and occlusion [10,11]. HUS is a form of TMA defined as a triad of intravascular hemolysis, thrombocytopenia, and acute renal failure [12]. HUS is labeled as typical when caused by Shiga toxin produced by *E. coli* O157:H7 or S. dysenteriae and is labeled atypical when caused by mutations or autoantibodies activating complement system [12]. Around 10% of HUS cases in children and the majority of the adult cases fall in the category of aHUS. Complement gene pathway mutations are recognized in only 50-60% of cases of aHUS, whereas the rest of the cases have impaired diacylglycerol kinase ε (DGKε) activity, cobalamin C deficiency, or plasminogen deficiency. *Clostridium difficile* infection is a rare precipitant of aHUS. Our patient was positive for *C. difficile* toxin A by EIA. Test for SLT by EIA was negative. Several treatment options are described in cases of *C. difficile*-associated aHUS, including steroids, antibiotics, and plasma exchange with favorable results [7]. Terminal complement inhibitors have emerged as an effective therapy for aHUS [13]. Eculizumab controls hemolysis and improves renal function [14]. Renal transplant in patients with aHUS is not recommended due to graft failure and high recurrence rate [15]. Patients with no previous history of aHUS also develop de novo aHUS after renal transplant [15]. Around 50% of patients with aHUS progress to end-stage renal disease [16]. Our patient responded positively with normalization of hemoglobin, platelet count, LDH, and creatinine levels. The use of eculizumab leads to the resolution of symptoms in patients with *C. difficile*-associated aHUS [9].

## Conclusions

aHUS carries a poor prognosis, and the mortality rate is high because most of the patients are diagnosed late in the disease course. *Clostridium difficile* is a rare precipitant of aHUS. Depending on the stage of the disease, treatment options include plasma exchange, eculizumab, and/or dialysis. Plasma exchange therapy is the most common intervention. Most of the patients require renal replacement therapy in the form of dialysis or renal transplant. Eculizumab is an effective therapy for the resolution of hemolysis and normalizing renal function.

## References

[1] Barbour T, Johnson S, Cohney S, Hughes P (2012). Thrombotic microangiopathy and associated renal disorders. Nephrol Dial Transplant.

[2] Atkinson JP, Liszewski MK, Richards A, Kavanagh D, Moulton EA (2005). Hemolytic uremic syndrome: an example of insufficient complement regulation on self-tissue. Ann N Y Acad Sci.

[3] Salvadori M, Bertoni E (2013). Update on hemolytic uremic syndrome: diagnostic and therapeutic recommendations. World J Nephrol.

[4] Noris M, Remuzzi G (2009). Atypical hemolytic-uremic syndrome. N Engl J Med.

[5] Ruggenenti P, Noris M, Remuzzi G (2001). Thrombotic microangiopathy, hemolytic uremic syndrome, and thrombotic thrombocytopenic purpura. Kidney Int.

[6] Kavanagh D, Goodship TH, Richards A (2006). Atypical haemolytic uraemic syndrome. Br Med Bull.

[7] Mogyorosi A, Carley MD. (1997). Hemolytic-uremic syndrome associated with pseudomembranous colitis caused by Clostridium difficile. Nephron.

[8] Keshtkar-Jahromi M, Mohebtash M (2012). Hemolytic uremic syndrome and Clostridium difficile colitis. J Community Hosp Intern Med Perspect.

[9] Inglis JM, Barbara JA, Juneja R, Milton C, Passaris G, Li JYZ Atypical haemolytic uraemic syndrome associated with Clostridium difficile infection successfully treated with eculizumab. Case Rep Nephrol.

[10] Benz K, Amann K (2010). Thrombotic microangiopathy: new insights. Curr Opin Nephrol Hypertens.

[11] Kerr H, Richards A (2012). Complement-mediated injury and protection of endothelium: lessons from atypical haemolytic uraemic syndrome. Immunobiology.

[12] Noris M, Remuzzi G (2005). Hemolytic uremic syndrome. J Am Soc Nephrol.

[13] Menne J, Delmas Y, Fakhouri F (2019). Outcomes in patients with atypical hemolytic uremic syndrome treated with eculizumab in a long-term observational study. BMC Nephrol.

[14] Walle JV, Delmas Y, Ardissino G, Wang J, Kincaid JF, Haller H (2017). Improved renal recovery in patients with atypical hemolytic uremic syndrome following rapid initiation of eculizumab treatment. J Nephrol.

[15] Gonzalez Suarez ML, Thongprayoon C, Mao MA, Leeaphorn N, Bathini T, Cheungpasitporn W (2019). Outcomes of kidney transplant patients with atypical hemolytic uremic syndrome treated with eculizumab: a systematic review and meta-analysis. J Clin Med.

[16] Afshar-Kharghan V (2016). Atypical hemolytic uremic syndrome. Hematology Am Soc Hematol Educ Program.

